# The BCS Energy Gap at High Density

**DOI:** 10.1007/s10955-022-02965-9

**Published:** 2022-07-29

**Authors:** Joscha Henheik, Asbjørn Bækgaard Lauritsen

**Affiliations:** IST Austria, Am Campus 1, 3400 Klosterneuburg, Austria

**Keywords:** BCS theory, Energy gap, Superconductivity, 81Q10, 46N50, 82D55

## Abstract

We study the BCS energy gap $\Xi$ in the high–density limit and derive an asymptotic formula, which strongly depends on the strength of the interaction potential *V* on the Fermi surface. In combination with the recent result by one of us (Math. Phys. Anal. Geom. 25, 3, 2022) on the critical temperature $T_c$ at high densities, we prove the universality of the ratio of the energy gap and the critical temperature.

## Introduction and Main Results

The Bardeen–Cooper–Schrieffer (BCS) theory [2] (see [11] for a review of recent rigorous mathematical work) has been an important theory of superconductivity since its conception. More recently, it has also gained attraction for describing the phenomenon of superfluidity in ultra cold fermionic gases, see [3, 4] for reviews. In either context, BCS theory is often formulated in terms of the BCS gap equation (at zero temperature)

1 $$\begin{aligned} \Delta (p) = -\frac{1}{(2\pi )^{3/2}} \int \limits _{\mathbb {R}^3} \hat{V}(p-q) \frac{\Delta (q)}{E_{\Delta ,\mu }(q)} \mathrm {d}q, \end{aligned}$$

where $E_{\Delta ,\mu }(p) = \sqrt{(p^2-\mu )^2+ \vert \Delta (p) \vert ^2}$. At finite temperature $T > 0$ one replaces $E_{\Delta , \mu }$ by $E_{\Delta ,\mu }/\tanh ( E_{\Delta , \mu } / 2T)$. The function $\Delta$ is interpreted as the order parameter describing the Cooper pairs (paired fermions). The interaction is local and given by the potential *V*, which we will assume to be satisfying $V \in L^1(\mathbb {R}^3)$, in which case it has a Fourier transform given by $(\mathfrak {F}V)(p):= \hat{V} (p) :=(2\pi )^{-3/2} \int _{\mathbb {R}^3} V(x) {\mathrm {e}}^{- \mathrm {i}p\cdot x} \,\text {d}x$.

The chemical potential $\mu$ controls the density of the fermions, and we investigate the high–density limit, i.e. $\mu \rightarrow \infty$, here. Recently this limit was studied by one of us [14], where an asymptotic formula for the critical temperature $T_c$ was found. For temperatures *T* below the critical temperature, $T < T_c$, the gap equation at temperature *T* (Eq. (1) with $E_{\Delta , \mu }$ replaced as prescribed) admits a non–trivial solution, for $T \ge T_c$ it does not. The critical temperature may equivalently be characterized by the existence of a negative eigenvalue of a certain linear operator, see [12]. Physically, a system at temperature *T* is superconducting/fluid if $T < T_c$, if $T \ge T_c$ it is not.

In this paper we study the energy gap (at zero temperature)

2 $$\begin{aligned} \Xi = \inf _p E_{\Delta ,\mu }(p) = \inf _p \sqrt{(p^2-\mu )^2+ \vert \Delta (p)\vert ^2}\,. \end{aligned}$$

The function $E_{\Delta ,\mu }$ has the interpretation of the dispersion relation for the corresponding BCS Hamiltonian, and so $\Xi$ is indeed an energy gap (see Appendix A in [12]). We show that, in the high–density limit, $\mu \rightarrow \infty$, the ratio of the energy gap and the critical temperature tends to a universal constant independent of the interaction potential,

3 $$\begin{aligned} \frac{\Xi }{T_c} \approx \frac{\pi }{{\mathrm {e}}^{\gamma }}\,, \end{aligned}$$

where $\gamma \approx 0.577$ denotes the Euler–Mascheroni constant. This universality is well–known in the physics literature, see, e.g., [7], and was rigorously verified in the weak–coupling limit by Hainzl and Seiringer [9] and in the low–density limit, $\mu \rightarrow 0$, by one of us [17] building on a work by Hainzl and Seiringer [8]. The general strategy for proving the universality in these limits has been to establish sufficiently good asymptotic formulas for both, $T_c$ and $\Xi$, and compare them afterwards.

The weak–coupling limit is studied in [6, 9], where one considers a potential $\lambda V$ for *V* fixed and a small coupling constant $\lambda \rightarrow 0$. In this limit, Hainzl and Seiringer [9] have shown that the critical temperature and energy gap satisfies $T_c \sim A \exp (-B/\lambda )$ and $\Xi \sim C \exp (-B/\lambda )$ respectively for explicit constants $A,B,C > 0$ depending on the interaction potential *V* and the chemical potential $\mu$. This limit exhibits the same universality and the ratio $C/A = \pi {\mathrm {e}}^{-\gamma }$ is independent of the interaction potential *V* and the chemical potential $\mu$.

The low–density limit $\mu \rightarrow 0$ is studied in [8, 17]. In this limit Hainzl and Seiringer [8] have shown that the critical temperature satisfies $T_c \sim \mu A \exp (-B/\sqrt{\mu })$ and one of us [17] has shown that the energy gap satisfies $\Xi \sim \mu C \exp (-B/\sqrt{\mu })$, for some (different) explicit constants $A,B,C > 0$ depending on the interaction potential *V*. Also in this limit we have the same universality and the ratio $C/A = \pi {\mathrm {e}}^{-\gamma }$ is independent of the interaction potential *V*. These results together with the present paper thus show that the universality (3) holds in both, the low– and high–density limit, as well as in the weak–coupling limit.

To show the universality, we prove in Theorem 3 an asymptotic formula for the energy gap $\Xi$ in the high–density limit, similar to the corresponding formula for the critical temperature given in Theorem 7 in [14]. This formula, as well as the one given in Theorem 3, depends strongly on the strength of the interaction potential *V* on the Fermi sphere $\{p^2 = \mu \}$, which becomes weak due to the decay of $\hat{V}$ in momentum space. Together with the formula for the critical temperature [14] we prove the universality (3) in Corollary 5. All proofs are given in Sect. 2. We now introduce some technical constructions and give the precise statements of our results.

### Preliminaries

We will work with the formulation of BCS theory of [6, 8, 9, 11, 12, 14, 17]. There one considers minimizers of the BCS functional (at zero temperature)

4 $$\begin{aligned} \mathcal {F}(\alpha ) = \frac{1}{2} \int \limits _{\mathbb {R}^3} |p^2 - \mu | \left( 1 - \sqrt{1 - 4|\hat{\alpha }(p)|^2}\right) \,\text {d}p + \int \limits _{\mathbb {R}^3} V(x) |\alpha (x)|^2 \,\text {d}x\,. \end{aligned}$$

If $\alpha$ is a minimizer of this, then $\Delta = -2\widehat{V\alpha }$ satisfies the BCS gap Eq. (1). As discussed in [9] the minimizer $\alpha$ is in general not necessarily unique, hence also $\Delta$ and $\Xi$ are not necessarily unique. However, since we will assume that the interaction *V* has non–positive Fourier transform, $\alpha$ and thus $\Xi$ is unique (see Lemma 2 in [9]).

A crucial role for the investigation of the energy gap (2) in the high–density limit is played by the (rescaled) operator $\mathcal {V}_{\mu } : L^2(\mathbb {S}^{2}) \rightarrow L^2(\mathbb {S}^{2})$ measuring the strength of the interaction potential $\hat{V}$ on the Fermi surface. It is defined as

5 $$\begin{aligned} \left( \mathcal {V}_{\mu } u\right) (p) = \frac{1}{(2\pi )^{3/2}} \int \limits _{ \mathbb {S} ^{2}} \hat{V}(\sqrt{\mu } (p-q)) u(q)\, \mathrm {d}\omega (q) \,, \end{aligned}$$

where $\mathrm {d}\omega$ denotes the uniform (Lebesgue) measure on the unit sphere $\mathbb {S} ^2$. The pointwise evaluation of $\hat{V}$ (and in particular on a $\mathrm {codim-}1$ submanifold) is well defined since $V \in L^1(\mathbb {R}^3)$. The condition that $V \in L^1(\mathbb {R}^3)$ could potentially be relaxed, see [5] and Remark 9 in [14]. The lowest eigenvalue of $\mathcal {V}_{\mu }$, which we denote by

$$\begin{aligned} e_{\mu } = \mathrm {inf\, spec}\, \mathcal {V}_{\mu } \end{aligned}$$

will be of particular importance. Note, that $\mathcal {V}_{\mu }$ is a trace–class operator (see the argument above Equation (3.2) in [6]) with

$$\begin{aligned} \mathrm {tr}(\mathcal {V}_{\mu }) = \frac{1}{2\pi ^2} \int \limits _{\mathbb {R}^3} V(x) \mathrm {d}x = \sqrt{\frac{2}{\pi }} \, \hat{V}(0)\,. \end{aligned}$$

We will assume that $\hat{V}(0) < 0$ in which case $e_{\mu } < 0$. This corresponds to an attractive interaction between (some) electrons on the Fermi sphere.

In this work, we restrict ourselves to the special case of radial potentials *V*, where the spectrum of $\mathcal {V}_{\mu }$ can be determined more explicitly (see, e.g., Sect. 2.1 in [6]). Indeed, for radial *V*, the eigenfunctions of $\mathcal {V}_{\mu }$ are spherical harmonics and the corresponding eigenvalues are

6 $$\begin{aligned} \frac{1}{2\pi ^2} \int \limits _{\mathbb {R}^3} V(x) \left( j_\ell (\sqrt{\mu }\vert x \vert )\right) ^2 \mathrm {d}x\,. \end{aligned}$$

The lowest eigenvalue $e_\mu$ is thus given by

$$\begin{aligned} e_\mu = \frac{1}{2\pi ^2} \, \inf _{\ell \in \mathbb {N}_0} \, \int \limits _{\mathbb {R}^3} V(x) \left( j_\ell (\sqrt{\mu }\vert x \vert )\right) ^2 \mathrm {d}x \,. \end{aligned}$$

Here, $j_\ell$ denotes the spherical Bessel function of order $\ell \in \mathbb {N}_0$. Additionally, in case that $\hat{V} \le 0$, we have, by the Perron–Frobenius theorem, that the minimal eigenvalue is attained for the constant eigenfunction (i.e. with $\ell = 0$). Thus

7 $$\begin{aligned} e_{\mu } = \frac{1}{2\pi ^2} \int _{\mathbb {R}^3} V(x) \left( \frac{\sin (\sqrt{\mu }\vert x \vert )}{\sqrt{\mu }\vert x \vert }\right) ^2 \mathrm {d}x\,. \end{aligned}$$

For further discussions of the radiality assumption on *V*, see Remark 8 in [14].

In order to obtain an asymptotic formula for the energy gap that is valid up to second order (see [9, 14]), we define the operator $\mathcal {W}_\mu ^{(\kappa )}$ on $u \in L^2( \mathbb {S} ^2)$ via its quadratic form

8 $$\begin{aligned} \big \langle u \big \vert \mathcal {W}_\mu ^{(\kappa )} \big \vert u \big \rangle&= \sqrt{\mu }\int \limits _{0}^{\infty } \mathrm {d}\vert p \vert \left( \frac{\vert p \vert ^2}{\vert \vert p \vert ^2 - 1 \vert } \left[ \int \limits _{ \mathbb {S} ^{2}}\mathrm {d}\omega (p) \left( \vert \hat{\varphi }(\sqrt{\mu } p) \vert ^2 - \vert \hat{\varphi }(\sqrt{\mu } p/\vert p \vert ) \vert ^2\right) \right] \right. \nonumber \\&\quad + \left. \frac{\vert p\vert ^2}{\vert p\vert ^2 + \kappa ^2} \int \limits _{ \mathbb {S} ^{2}} \mathrm {d}\omega (p) \vert \hat{\varphi }(\sqrt{\mu } p/\vert p \vert ) \vert ^2 \right) \end{aligned}$$

for any fixed $\kappa \ge 0$ (cf. Eq. (10) in [14] resp. Equation (13) in [9] for an analogous definition with $\kappa = 0$). Here $\hat{\varphi }(p) = (2\pi )^{-3/2} \int _{ \mathbb {S} ^{2}} \hat{V}(p-\sqrt{\mu }q) u(q) \mathrm {d}\omega (q)$, and $(\vert p\vert , \omega (p)) \in (0,\infty ) \times \mathbb {S} ^2$ denote spherical coordinates for $p \in \mathbb {R}^3$. To see that this operator is well–defined note that the map $\vert p \vert \mapsto \int _{ \mathbb {S} ^{2}} \mathrm {d}\omega (p) \vert \hat{\varphi }(p)\vert ^2$ is Lipschitz continuous for any $u \in L^2( \mathbb {S} ^2)$ since $V \in L^1(\mathbb {R}^3)$. Hence the radial integral in Eq. (8) is well defined for $\vert p\vert \sim 1$. We will further assume that $V \in L^{3/2}(\mathbb {R}^3)$, in which case the integral is well–defined for large $\vert p \vert$ as well. We formulate our result in Theorem 3 only for $\kappa = 0$, but the case of a positive parameter $\kappa >0$ is crucial in the proof of this statement. For example, $\kappa >0$ ensures that the second term in the decomposition of the Birman–Schwinger operator associated with $E_{\Delta ,\mu } + V$ is small (cf. Eq. (14)). Whenever it does not lead to confusion, we will refer to some $\kappa$-dependent quantity at $\kappa = 0$ by simply dropping the $(\kappa )$-superscript.

We now define the operator

9 $$\begin{aligned} \mathcal {B}_\mu ^{(\kappa )} = \frac{\pi }{2}\left( \mathcal {V}_\mu - \mathcal {W}_\mu ^{(\kappa )}\right) \,, \end{aligned}$$

which captures the strength of the interaction potential near the Fermi surface to second order and denote its lowest eigenvalue by

10 $$\begin{aligned} b_\mu ^{(\kappa )} = \mathrm {inf \, spec} \, \mathcal {B}_\mu ^{(\kappa )}\,. \end{aligned}$$

The factor $\pi /2$ is introduced in Eq. (9) since for this scaling, the eigenvalue $b_\mu ^{(\kappa )}$ has the interpretation of an effective scattering length in the case of small $\mu$ (see Proposition 1 in [9]). Moreover, it was shown during the proof of Theorem 7 in [14] that if $e_\mu <0$ then also $b_\mu ^{(\kappa )}<0$ for $\mu$ large enough. This will also follow from Eq. (29) in the proof below.

### Results

The following definition characterizes the class of interaction potentials for which our asymptotic formula will hold.

#### Definition 1

(*Admissible potentials*) Let $V \in L^1(\mathbb {R}^3) \cap L^{3/2}(\mathbb {R}^3)$ be a radial real–valued function with non–positive Fourier transform $\hat{V} \le 0$ and $\hat{V}(0) <0$. Denote

11 $$\begin{aligned} s_\pm ^* := \sup \left\{ s \ge 0 : |\cdot |^{-s} V_\pm \in L^1(\mathbb {R}^3)\right\} , \qquad s^* := \min \{s_+^*, s_-^*\}\, , \end{aligned}$$

where $V_\pm = \max \{ \pm V, 0\}$ are the positive and negative parts of *V*. We say that *V* is *admissible* if the following is satisfied:

- There exists $a > 0$ such that
  $$\begin{aligned} \sup \left\{ r \ge 0 : \lim _{\varepsilon \rightarrow 0} \frac{1}{\varepsilon ^r}\int \limits _{B_\varepsilon } V_\pm (x) \,\text {d}x = 0\right\} = \sup \left\{ r \ge 0 : \lim _{\varepsilon \rightarrow 0} \frac{1}{\varepsilon ^r} \int \limits _{B_\varepsilon } V_\pm |_{B_{a}}^*(x) \,\text {d}x = 0 \right\} \!, \end{aligned}$$
  where $V_\pm |_{B_{a}}^*$ denotes the symmetric decreasing rearrangement of $V_\pm |_{B_{a}}$, the restriction of $V_\pm$ to the ball of radius *a* around 0,
- if $|\cdot |^{-2}V \notin L^1(\mathbb {R}^3)$, we have $s^* = s_-^* < s^*_+$, and
- $$\vert \cdot \vert V \in L^2(\mathbb {R}^3)$$ and $$s^* > 7/5$$.

As discussed around Eq. (4), the definiteness of the Fourier transform is needed for ensuring uniqueness of the energy gap $\Xi$. Intuitively, the other criteria may be though as follows: Assumption (a) captures that the strongest singularity of *V* near the origin is in fact at the origin, assumption (b) captures that *V* is predominantly attractive, and assumption (c) captures that *V* is slightly less divergent at the origin, than allowed by the $L^{3/2}(\mathbb {R}^3)$-assumption. In view of assumption (a), we remark that it is natural that the system is sensitive to the short range behavior of the interaction potential, since the interparticle distance as the physically relevant length scale that depends on the particle density tends to zero in the high–density limit. Furthermore, note that for $V \in L^1(\mathbb {R}^3) \cap L^{3/2}(\mathbb {R}^3)$, the condition $\vert \cdot \vert V \in L^2(\mathbb {R}^3)$ is mainly about regularity away from zero and infinity.

The most important examples of allowed interaction potentials include the cases of attractive Gaussian, Lorentzian and Yukawa potentials, also discussed in [16]. That is

$$\begin{aligned} V_{\text {Gauss}}(x) = - (2\pi )^{-3/2}{\mathrm {e}}^{-x^2/2}\,, \quad V_{\text {Lorentz}}(x) = - \frac{1}{\pi ^2(1 + x^2)^2}\,, \quad V_{\text {Yukawa}}(x) = - \frac{1}{4\pi |x|}{\mathrm {e}}^{-|x|}\,. \end{aligned}$$

#### Remark 2

The proof of our main result formulated in Theorem 3 works without change if we assume $|\cdot |V\in L^r(\mathbb {R}^3)$ for some $2 \ge r > f(s^*)$ instead of $|\cdot |V\in L^2(\mathbb {R}^3)$, where *f* is some complicated (explicit) expression, see the proof of Proposition 14. We do not state the theorem with this slight generalization for simplicity. We will however give the proof under this more general assumption for the purpose of illuminating where the assumption on $r=2$ comes from. Additionally, to further illuminate where the conditions are used, all propositions and lemmas are stated with only the conditions needed on *V* for that specific statement. (Beyond the conditions that $V\in L^1(\mathbb {R}^3)\cap L^{3/2}(\mathbb {R}^3)$ is real–valued, radial and has $\hat{V} \le 0, \hat{V}(0) < 0$, which is always assumed.)

We can now state our main result for admissible interaction potentials.

#### Theorem 3

Let *V* be an admissible potential. Then the energy gap $\Xi$ is positive and satisfies

12 $$\begin{aligned} \lim \limits _{\mu \rightarrow \infty } \left( \log \frac{\mu }{\Xi } + \frac{\pi }{2\sqrt{\mu } b_\mu }\right) = 2- \log (8)\,. \end{aligned}$$

In other words,

$$\begin{aligned} \Xi = \mu \left( 8\, {\mathrm {e}}^{-2} + o(1)\right) \exp \left( \frac{\pi }{2 \sqrt{\mu } b_\mu }\right) \end{aligned}$$

in the limit $\mu \rightarrow \infty$. Similarly as for the critical temperature [14], this asymptotic formula is completely analogous to the weak–coupling case [9] (replace $V \rightarrow \lambda V$ and take the limit $\lambda \rightarrow 0$) but we have coupling parameter $\lambda =1$ here. This similarity is not entirely surprising. From a physical perspective, only those fermions with momenta close to the Fermi surface $\{p^2 = \mu \}$ contribute to the superconductivity/fluidity. Thus, by the decay of the interaction $\hat{V}$ in Fourier space, the high–density limit, $\mu \rightarrow \infty$, is effectively a weak–coupling limit.

In order to deduce universality as in Eq. (3) in the high–density limit, we show that every admissible potential in the sense of Definition 1 satisfies the imposed conditions for the proof of an analogous formula for the critical temperature. These conditions were formulated in Definition 5 in [14].

#### Proposition 4

Every admissible potential satisfies the conditions of Definition 5 in [14].

#### Proof

By comparing the two definitions, the statement is trivial apart from the following two points. First, the additional requirement $\int _{\mathbb {R}^3} \frac{V(x)}{\vert x \vert ^2} \mathrm {d}x < 0$ from Definition 5 in [14] in the case $|\cdot |^{-2}V \in L^1(\mathbb {R}^3)$ is automatically fulfilled, since

$$\begin{aligned} -\Delta _p \widehat{\frac{V}{\vert \cdot \vert ^2}}(p) = \hat{V}(p) \le 0\,. \end{aligned}$$

That is, the radial function $\widehat{\frac{V}{\vert \cdot \vert ^2}}$ is subharmonic and approaches 0 as $\vert p \vert \rightarrow \infty$ (by the Riemann–Lebesgue Lemma), and thus by the maximum principle assumes a strictly negative value at 0. Second, since $\hat{V} \le 0$ and by application of the Perron–Frobenius Theorem, the constant spherical harmonic is the unique normalized ground state of $\mathcal {V}_\mu$ and thus condition (d) from Definition 5 in [14] can be dropped. $\square$

Therefore, by means of Theorem 7 in [14], the critical temperature $T_c$ satisfies

$$\begin{aligned} T_c = \mu \left( \frac{8}{\pi }{\mathrm {e}}^{\gamma -2} + o(1)\right) \exp \left( \frac{\pi }{2 \sqrt{\mu } b_\mu }\right) \end{aligned}$$

for any admissible potential. Here $\gamma \approx 0.577$ is the Euler–Mascheroni constant. Together with Theorem 3, this immediately proves the following.

#### Corollary 5

Let *V* be an admissible potential. Then

$$\begin{aligned} \lim \limits _{\mu \rightarrow \infty } \frac{\Xi }{T_c} = \frac{\pi }{{\mathrm {e}}^\gamma } \approx 1.764\,. \end{aligned}$$

This universality of the ratio between the energy gap and the critical temperature is well known in the physics literature (see, e.g., [7]) and has been previously established rigorously in the weak–coupling and low–density limits (see [9] resp. [17]).

## Proofs

As in the analysis of the critical temperature [14] we introduce the parameter $\kappa >0$. We have the following comparison of $b_\mu ^{(\kappa )}$ with the $\kappa = 0$ quantity.

### Lemma 6

[14,  Lemma 15] Let *V* be admissible and $\kappa > 0$. In the limit of high density, $\mu \rightarrow \infty$, we have

$$\begin{aligned} \frac{\pi }{2\sqrt{\mu } b_\mu } = \frac{\pi }{2\sqrt{\mu } b_\mu ^{(\kappa )}} + \kappa \, \frac{\pi }{2} + o(1)\,. \end{aligned}$$

### Proof

This is immediate from Lemma 15 in [14] by invoking Proposition 4. $\square$

Now, one important ingredient in our proof is the asymptotic behavior of

$$\begin{aligned} m_{\mu }^{(\kappa )}(\Delta ) = \frac{1}{4\pi } \int \limits _{\mathbb {R}^3} \left( \frac{1}{E_{\Delta ,\mu }(p)} - \frac{1}{p^2+\kappa ^2\mu }\right) \mathrm {d}p \end{aligned}$$

for fixed $\kappa > 0$ (recall that $E_{\Delta ,\mu }(p) = \sqrt{(p^2-\mu )^2+\vert \Delta (p)\vert ^2}$). This is similar to the strategy for the weak–coupling, low–density, and high–density limits of the critical temperature (see [8, 9, 14]), and for the weak–coupling and low–density limits of the energy gap (see [9, 17]).

### Lemma 7

Let *V* be admissible and $\kappa > 0$. In the limit of high density, $\mu \rightarrow \infty$, we have

$$\begin{aligned}&\Xi = \Delta (\sqrt{\mu })(1+o(1))\,, \\&m_\mu ^{(\kappa )}(\Delta ) = \sqrt{\mu } \left( \log \frac{\mu }{\Delta (\sqrt{\mu })}-2+\kappa \, \frac{\pi }{2} + \log (8) + o(1) \right) \,,\\&\frac{m_\mu ^{(\kappa )}(\Delta )}{\sqrt{\mu }} = - \frac{\pi }{2\sqrt{\mu }b_\mu ^{(\kappa )}} + o(1)\,. \end{aligned}$$

These three asymptotic equalities are proven in Propositions 10, 14, and 15 respectively.

### Proof of Theorem 3

By Lemmas 7 and 6 we get

$$\begin{aligned} \lim \limits _{\mu \rightarrow \infty } \left( \log \frac{\mu }{\Xi } + \frac{\pi }{2\sqrt{\mu }b_\mu }\right)&= \lim \limits _{\mu \rightarrow \infty } \left( \log \frac{\mu }{\Delta (\sqrt{\mu })} + \frac{\pi }{2\sqrt{\mu }b_\mu }\right) \\&= \lim \limits _{\mu \rightarrow \infty } \left( \log \frac{\mu }{\Delta (\sqrt{\mu })} + \frac{\pi }{2\sqrt{\mu }b_\mu ^{(\kappa )}}\right) + \kappa \, \frac{\pi }{2}\\&= \lim \limits _{\mu \rightarrow \infty } \left( \log \frac{\mu }{\Delta (\sqrt{\mu })} - \frac{m_\mu ^{(\kappa )}(\Delta )}{\sqrt{\mu }}\right) + \kappa \, \frac{\pi }{2} \\&= \ 2-\kappa \, \frac{\pi }{2} + \log (8)+ \kappa \, \frac{\pi }{2} = 2-\log (8)\,, \end{aligned}$$

which yields (12) and we have proven Theorem 3. $\square$

The rest of this paper is devoted to the proof of Lemma 7.

### Proof of Lemma 7

As remarked, a key idea is to study the integral $m_\mu ^{(\kappa )}(\Delta )$. As in [9, 17] we first need some control of $\Delta$ in the form of a Lipschitz–like bound (given in Lemma 9) and a bound controlling $\Delta (p)$ in terms of $\Delta (\sqrt{\mu }q)$ for $q\in \mathbb {S} ^2$ (given in Equation (22)). First, we recall some properties (from [9]) of the minimizer $\alpha$ of the BCS functional at zero temperature

13 $$\begin{aligned} \mathcal {F}(\alpha ) = \frac{1}{2} \int \limits _{\mathbb {R}^3}|p^2 - \mu | \left( 1 - \sqrt{1 - 4|\hat{\alpha }(p)|^2}\right) \,\text {d}p + \int \limits _{\mathbb {R}^3} V(x) |\alpha (x)|^2 \,\text {d}x\,. \end{aligned}$$

In [9,  Lemma 2] it is shown that for potentials *V* with non–positive Fourier transform there exists a unique minimizer $\alpha$ with (strictly) positive Fourier transform. Moreover, for radial *V* the BCS functional is invariant under rotations. Hence $\alpha$ and thus also $\Delta = -2\widehat{V\alpha }$ are radial functions. Therefore, with a slight abuse of notation, we will write $\Delta (|p|)$ and mean $\Delta (p)$ for some (any) vector *p*. (In general for any radial function *f*, we will write *f*(|*p*|) for the value of *f*(*p*).) Additionally, since $\hat{V}\le 0$ we have that $\Delta \ge 0$. In fact, by the BCS gap equation (1), we even have $\Delta > 0$, see Lemma 2 in [9]. Now, we give some *a priori* bounds on the minimizer $\alpha$. The proofs of Lemmas 8 and 9 are given in Sect. 2.2.

#### Lemma 8

Let $\alpha$ be the minimizer of the BCS functional (13). Then for large $\mu$

$$\begin{aligned} \left\| \alpha \right\| _{L^2}\le C \mu ^{7/20} \quad \text {and} \quad \left\| \alpha \right\| _{H^1} \le C \mu ^{3/4}. \end{aligned}$$

These estimates on the minimizer $\alpha$ now translate to bounds on $\Delta = -2\widehat{V\alpha }$.

#### Lemma 9

Suppose $V\in L^{r}(\mathbb {R}^3)$ for some $6/5 \le r \le 2$. Define $\delta _r = \frac{3}{4} - \frac{6}{5r}$. Then for sufficiently large $\mu$ we have

$$\begin{aligned} \left\| \Delta \right\| _{L^\infty } \le C \mu ^{\frac{24-5r}{20r}} = C\mu ^{\frac{1}{2} - \delta _r}. \end{aligned}$$

Similarly, if $|\cdot | V \in L^{r}(\mathbb {R}^3)$ then

$$\begin{aligned} |\Delta (p) - \Delta (q)| \le C \mu ^{\frac{24-5r}{20r}} ||p| -|q|| = C\mu ^{\frac{1}{2} - \delta _r}||p|-|q|| \end{aligned}$$

for all *p*, *q*. In particular, if $r > 8/5$ then $\delta _r > 0$ and thus $1/2-\delta _r< 1/2$.

We will use the first bound as $\Vert \Delta \Vert _{L^\infty } \le C \mu ^{11/20} = o(\mu )$ for $r = 3/2$, and the second bound as $\vert \Delta (p) - \Delta (q) \vert \le C \mu ^{7/20} \vert \vert p\vert - \vert q \vert \vert$ for $r=2$.

Armed with these *a priori* bounds on $\Delta$, we can now prove the asymptotic formulas in Lemma 7 and start with the first one.

#### Proposition 10

Suppose $|\cdot | V \in L^r({\mathbb {R}^3})$ for $r > 8/5$. Then $\Xi = \Delta (\sqrt{\mu }) (1 + o(1))$.

#### Proof

Clearly $\Xi = \inf \sqrt{|p^2 - \mu |^2 + |\Delta (p)|^2}\le \Delta (\sqrt{\mu })$. Take now $p \in \mathbb {R}^3$ with $|p^2- \mu | \le \Xi \le \Delta (\sqrt{\mu })$. Then

$$\begin{aligned} |\Delta (p) - \Delta (\sqrt{\mu })| \le C \mu ^{1/2 -\delta _r} ||p|-\sqrt{\mu }| \le C \mu ^{1/2 - \delta _r} \frac{\Delta (\sqrt{\mu })}{|p| + \sqrt{\mu }} \le C \mu ^{-\delta _r} \Delta (\sqrt{\mu }) \end{aligned}$$

where $\delta _r >0$ by assumption. Hence, $\Delta (p) = \Delta (\sqrt{\mu })(1 + o(1))$ for any such *p* and we conclude the desired. $\square$

The proofs of the second and third equality (Propositions 14 and 15, respectively) heavily use Lemmas 11 and 12, which we import from [14]. Lemma 11 provides an upper bound for integrals of the potential against spherical Bessel functions $j_\ell$, uniformly in $\ell \in \mathbb {N}_0$. These naturally arise by the spherical symmetry of *V* (cf.Eq. (6)).

#### Lemma 11

([14,  Lemma 12]) Let $V \in L^1(\mathbb {R}^3) \cap L^{3/2}(\mathbb {R}^3)$ and assume that $s^* >1$, with $s^*$ as in Definition 1. Set

$$\begin{aligned} \beta ^* = {\left\{ \begin{array}{ll} \frac{s^*}{2} &{} \text {for} \quad s^* \in (1,5/3]\\ \min \left( \frac{4s^*-4}{9s^*-7}+\frac{1}{2}, \, \frac{19}{22}\right) \quad &{} \text {for} \quad s^* >5/3 \,. \end{array}\right. } \end{aligned}$$

Note that $\beta ^*$ depends continuously on $s^*$ and is (strictly) monotonically increasing (between 1 and 2), and $\beta ^* \le \min (s^*,2)/2$ for any $s^*>1$. Then for any $\delta >0$ there exists an $\varepsilon _0 >0$ such that for all $\varepsilon \in [0,\varepsilon _0]$ we have

$$\begin{aligned} \limsup \limits _{\mu \rightarrow \infty } \mu ^{\beta ^* - \delta } \sup _{\ell \in \mathbb {N}_0}\int \limits _{\mathbb {R}^3} \mathrm {d}x \vert V (x)\vert \vert j_\ell (\sqrt{\mu }\vert x \vert )\vert ^{2-\varepsilon } = 0\,. \end{aligned}$$

Lemma 12 gives a lower bound on the quantity $e_\mu$ that measures the strength of the interaction potential on the Fermi surface (see Eq. (7)).

#### Lemma 12

([14,  Lemma 13]) Let *V* be an admissible potential (cf. Definition 1). Then for any $\delta >0$ there exists $c_\delta >0$ such that

$$\begin{aligned} \liminf \limits _{\mu \rightarrow \infty } \vert \mu ^{\min (s^*+ \delta ,2)/2} \, e_\mu \vert \ge c_\delta \,. \end{aligned}$$

#### Proof

This is immediate from Lemma 13 in [14] by invoking Proposition 4. $\square$

An upper bound is trivially obtained as $\vert e_\mu \vert \le C_\delta \mu ^{-\min (s^*-\delta ,2)/2}$ for any $\delta >0$ by definition of $s^*$ in Eq. (11) (see also Eq. (21)). Note that both, upper and lower bound, remain true if we replace the exponent with $\min (s^*,2)/2 \pm \delta$, i.e. $c_\delta \mu ^{-\min (s^*,2)/2 - \delta }\le |e_\mu | \le C_\delta \mu ^{-\min (s^*,2)/2 + \delta }$. This is the formulation we will use.

Beside these two Lemmas, we will use the following observation: It can easily be checked (see Lemma 3 in [9]) that the operator $E_{\Delta ,\mu }(p) + V(x)$ has 0 as its lowest eigenvalue, and that $\alpha$ is the (unique) eigenvector with this eigenvalue. By employing the Birman–Schwinger principle (see [6, 11, 12]), this is equivalent to the fact that the Birman–Schwinger operator

$$\begin{aligned} B_{\Delta ,\mu } = V^{1/2} \frac{1}{E_{\Delta ,\mu }} \vert V \vert ^{1/2} \end{aligned}$$

has $-1$ as its lowest eigenvalue with $V^{1/2}\alpha$ being the corresponding (unique) eigenvector. Here we used the notation $V(x)^{1/2} = \mathrm {sgn}(V(x)) \vert V(x)\vert ^{1/2}$. In the following we need a convenient decomposition of $B_{\Delta , \mu }$ in a dominant singular term and other error terms. For this purpose we let ${\mathfrak {F}_{\mu }} : L^1(\mathbb {R}^3) \rightarrow L^2( \mathbb {S} ^{2})$ denote the (rescaled) Fourier transform restricted to $\mathbb {S} ^{2}$ with

$$\begin{aligned} \left( \mathfrak {F}_{\mu } \psi \right) (p) = \frac{1}{(2\pi )^{3/2}} \int _{\mathbb {R}^3} {\mathrm {e}}^{-\mathrm {i} \sqrt{\mu } p\cdot x} \psi (x) \mathrm {d}x\,, \end{aligned}$$

which is well-defined by the Riemann–Lebesgue Lemma. Now, we decompose the Birman–Schwinger operator as

14 $$\begin{aligned} B_{\Delta ,\mu } = m_\mu ^{(\kappa )}(\Delta ) \, V^{1/2} {\mathfrak {F}_{\mu }}^\dagger {\mathfrak {F}_{\mu }} \vert V \vert ^{1/2} + V^{1/2} M^{(\kappa )}_{\Delta ,\mu }\vert V \vert ^{1/2} \,, \end{aligned}$$

where $M_{\Delta ,\mu }^{(\kappa )}$ is such that this holds. For the first term, note that $V^{1/2} {\mathfrak {F}_{\mu }} ^\dagger {\mathfrak {F}_{\mu }} \vert V \vert ^{1/2}$ is isospectral to $\mathcal {V}_\mu = {\mathfrak {F}_{\mu }} V {\mathfrak {F}_{\mu }}^\dagger$. In fact, the spectra agree at first except possibly at 0, but 0 is in both spectra as the operators are compact on an infinite dimensional space. This first term in the decomposition (14) will be the dominant term, which is how the third equality in Lemma 7 will arise.

Analogously to the proof of Lemma 14 in [14] and the proof of Theorem 1 in [9], we further decompose

15 $$\begin{aligned} V^{1/2} M^{(\kappa )}_{\Delta ,\mu }\vert V \vert ^{1/2} = V^{1/2} \frac{1}{p^2 + \kappa ^2 \mu }\vert V \vert ^{1/2} + A^{(\kappa )}_{\Delta ,\mu } =: L^{(\kappa )}_{\mu } + A^{(\kappa )}_{\Delta ,\mu }\,, \end{aligned}$$

where now $A^{(\kappa )}_{\Delta ,\mu }$ is such that this holds. In [14,  Eq. (29)] it was shown that

$$\begin{aligned} \left\| L^{(\kappa )}_{\mu } \right\| _{\mathrm {op}} \le C \, \mu ^{1/2} \, \int \limits _{0}^{\infty } \mathrm {d}p \frac{p^2}{p^2 + \kappa ^2} \sup _{\ell \in \mathbb {N} _0} \int \limits _{\mathbb {R}^3} \mathrm {d}x \vert V(x)\vert \, \vert j_\ell (\sqrt{\mu } p \vert x \vert ) \vert ^2, \end{aligned}$$

which may be bounded by $\mu ^{-\beta ^* + 1/2 + \delta }$ for any $\delta > 0$ by means of Lemma 11. We continue with a bound on the operator norm of $A_{\Delta , \mu }^{(\kappa )}$ by estimating the matrix elements $\langle f \vert A_{\Delta , \mu }^{(\kappa )} \vert g \rangle$ for functions $f,g\in L^2(\mathbb {R}^3)$. This computation is analogous to the computation in the proof of Theorem 2 in [14]. We give it here for completeness.

Note that, since *V* is radial, it is enough to restrict to functions of definite angular momentum. That is, with a slight abuse of notation, functions of the form $f(x) = Y_\ell ^m(\hat{x})f(|x|)$, where $Y_\ell ^m$ denotes the spherical harmonics and we write $\hat{x} = x/|x|$. The operator $A_{\Delta , \mu }^{(\kappa )}$ is indeed block–diagonal in the angular momentum as will follow from the computations below. Since functions of definite angular momentum span $L^2(\mathbb {R}^3)$ [13, Sects. 17.6–17.7] it is thus enough to bound $\langle f \vert A_{\Delta , \mu }^{(\kappa )} \vert g \rangle$ for *f*, *g* of the form $f(x) = Y_\ell ^m(\hat{x})f(|x|)$, $g(x) = Y_{\ell '}^{m'}(\hat{x})g(|x|)$.

Now, $A_{\Delta , \mu }^{(\kappa )}$ has integral kernel

$$\begin{aligned} A_{\Delta , \mu }^{(\kappa )}(x,y) = CV^{1/2}(x)|V(y)|^{1/2} \int \limits _{\mathbb {R}^3} \left( \frac{1}{E_{\Delta , \mu }(p)} - \frac{1}{p^2 + \kappa ^2\mu }\right) \left( {\mathrm {e}}^{\mathrm {i}p\cdot (x-y)} - {\mathrm {e}}^{\mathrm {i}\sqrt{\mu }\hat{p}\cdot (x-y)}\right) \,\text {d}p\,. \end{aligned}$$

Thus, by the radiality of *V* we get

16 $$\begin{aligned} \begin{aligned} \big \langle f \big \vert A_{\Delta , \mu }^{(\kappa )} \big \vert g \big \rangle&= C\int \limits _0^\infty \,\text {d}|x| \, |x|^2 V^{1/2}(|x|) \overline{f(|x|)} \int \limits _0^\infty \,\text {d}|y| \, |y|^2 |V(|y|)|^{1/2} g(|y|) \\&\quad \times \int _{0}^\infty \,\text {d}|p| \, |p|^2\left( \frac{1}{E_{\Delta , \mu }(|p|)} - \frac{1}{|p|^2 + \kappa ^2\mu }\right) \int \limits _{ \mathbb {S} ^2} \,\text {d}\omega (\hat{p}) \\&\quad \times \int \limits _{ \mathbb {S} ^2} \,\text {d}\omega (\hat{x}) \int _{ \mathbb {S} ^2}\,\text {d}\omega (\hat{y}) \, \overline{Y_\ell ^m(\hat{x})} Y_{\ell '}^{m'}(\hat{y}) \left( {\mathrm {e}}^{- \mathrm {i}p\cdot (x-y)} - {\mathrm {e}}^{- \mathrm {i}\sqrt{\mu }\hat{p}\cdot (x-y)}\right) \,. \end{aligned} \end{aligned}$$

Now, using the plane–wave expansion ${\mathrm {e}}^{\mathrm {i}p\cdot x} = 4\pi \sum _{\ell =0}^{\infty } \sum _{m= -\ell }^{\ell } \mathrm {i}^\ell j_\ell (|p||x|) Y_{\ell }^m(\hat{p}) \overline{Y_\ell ^m(\hat{x})}$, the spherical integrations in $\hat{x}$ and $\hat{y}$ may be evaluated as

$$\begin{aligned} 16\pi ^2 (-\mathrm {i})^{\ell + \ell '}\left( j_\ell (|p||x|) j_{\ell '}(|p||y|) - j_\ell (\sqrt{\mu }|x|)j_{\ell '}(\sqrt{\mu }|y|)\right) \overline{Y_{\ell }^m(\hat{p})} Y_{\ell '}^{m'}(\hat{p}) \end{aligned}$$

using the orthogonality of the spherical harmonics. The spherical $\hat{p}$-integral of this gives a factor $\delta _{\ell \ell '}\delta _{m m'}$ again by orthogonality of the spherical harmonics. (This shows that $A_{\Delta , \mu }^{(\kappa )}$ is block–diagonal in the angular momentum as claimed.) We may thus restrict to the case of $\ell = \ell '$ and $m=m'$. Hereinafter, we will write *x*, *y*, and *p* instead of |*x*|, |*y*|, and |*p*|.

Recall the following bounds on spherical Bessel functions

$$\begin{aligned} \sup _{\ell \in \mathbb {N} _0} \sup _{x\ge 0} |j_\ell (x)| \le 1\,, \qquad \sup _{\ell \in \mathbb {N} _0} \sup _{x\ge 0} |j_\ell '(x)| \le 1\,, \qquad \sup _{\ell \in \mathbb {N} _0} \sup _{x\ge 0} x^{5/6}|j_\ell (x)| \le C\,, \end{aligned}$$

where the first one is elementary, the second one follows from [1,  Eq. 10.1.20], and the third one may be found in [15,  Eq. 1] (see also Proposition 16 in [14]). Adding $\pm j_\ell (px)j_\ell (\sqrt{\mu }y)$ and using these bounds we may estimate for any $0< \varepsilon < 5/11$

17 $$\begin{aligned} \begin{aligned}&\left| j_\ell (px) j_{\ell }(py) - j_\ell (\sqrt{\mu }x)j_{\ell }(\sqrt{\mu }y)\right| \\&\qquad \le C |p - \sqrt{\mu }|^\varepsilon \left( p^{-\varepsilon } + (\sqrt{\mu })^{-\varepsilon }\right) \left( |j_\ell (px)|^{1-11\varepsilon /5} + |j_\ell (\sqrt{\mu }x)|^{1-11\varepsilon /5}\right) \\&\qquad \quad \times \left( |j_\ell (py)|^{1-11\varepsilon /5} + |j_\ell (\sqrt{\mu }y)|^{1-11\varepsilon /5}\right) . \end{aligned} \end{aligned}$$

The radial *p*–integral in Eq. (16) is then (a constant times)

18 $$\begin{aligned} \int \limits _{0}^\infty \,\text {d}p \left( \frac{1}{E_{\Delta , \mu }(p)} - \frac{1}{p^2 + \kappa ^2\mu }\right) \left( j_\ell (px) j_{\ell }(py) - j_\ell (\sqrt{\mu }x)j_{\ell }(\sqrt{\mu }y)\right) . \end{aligned}$$

Using Eq. (17) and changing the integration variable $p \rightarrow \sqrt{\mu }p$ we get

$$\begin{aligned} \begin{aligned} |18|&\le C\mu ^{1/2} \int \limits _0^\infty \,\text {d}p \, p^2 \left| \frac{1}{\sqrt{(p^2 - 1)^2 + \vert \Delta (\sqrt{\mu } p) /\mu \vert ^2 }}- \frac{1}{p^2 + \kappa ^2 }\right| \vert p-1\vert ^{\varepsilon } \left( \frac{1}{p^\varepsilon }+ 1\right) \\&\quad \times \left( |j_\ell (\sqrt{\mu }px)|^{1-11\varepsilon /5} + |j_\ell (\sqrt{\mu }x)|^{1-11\varepsilon /5}\right) \left( |j_\ell (\sqrt{\mu }py)|^{1-11\varepsilon /5} + |j_\ell (\sqrt{\mu }y)|^{1-11\varepsilon /5}\right) . \end{aligned} \end{aligned}$$

Plugging this into Eq. (16) and using Hölder’s inequality for the *x*- and *y*-integrations we thus get

$$\begin{aligned} \begin{aligned}&\big \vert \big \langle f \big \vert A_{\Delta , \mu }^{(\kappa )} \big \vert g \big \rangle \big \vert \\&\quad \le C \mu ^{1/2} \int \limits _{0}^{\infty } \mathrm {d}p \, p^2 \left| \frac{1}{\sqrt{(p^2 - 1)^2 + \vert \Delta (\sqrt{\mu } p) /\mu \vert ^2 }}- \frac{1}{p^2 + \kappa ^2 }\right| \vert p-1\vert ^{\varepsilon } \left( \frac{1}{p^\varepsilon }+ 1\right) \\&\qquad \times \int \limits _{\mathbb {R}^3} \mathrm {d}x \, \vert V (x)\vert \left( \vert j_\ell (\sqrt{\mu }p\vert x \vert ) \vert ^{2-22\varepsilon /5} + \vert j_\ell (\sqrt{\mu }\vert x \vert ) \vert ^{2-22\varepsilon /5}\right) \, \Vert f \Vert _{L^2} \, \Vert g \Vert _ {L^2}, \end{aligned} \end{aligned}$$

where we changed back to *x* denoting a vector in $\mathbb {R}^3$. By Lemma 11 we may bound the *x*-integral by $\mu ^{-\beta ^* + \delta }(1 + p^{-\beta ^* + \delta })$ for any $\delta > 0$. Also, $\Vert \Delta \Vert _{L^\infty } = o(\mu )$ by Proposition 9. Hence the *p*-integral will be finite uniformly in $\mu$ for $\mu$ large enough. We conclude that

$$\begin{aligned} \left\| A_{\Delta , \mu }^{(\kappa )} \right\| _{\text {op}} \le C \mu ^{-\beta ^* + 1/2 + \delta } \end{aligned}$$

for any $\delta > 0$ and for $\mu$ large enough. Combining this with the bound on $\Vert L_\mu ^{(\kappa )}\Vert _{\mathrm {op}}$ from above, we get

19 $$\begin{aligned} \limsup \limits _{\mu \rightarrow \infty } \mu ^{\beta ^*-1/2-\delta } \left\| V^{1/2} M_{\Delta ,\mu }^{(\kappa )} \vert V \vert ^{1/2} \right\| _{\mathrm {op}} = 0 \end{aligned}$$

for any $\delta >0$. Also, since $V^{1/2} \mathfrak {F}_\mu ^\dagger \mathfrak {F}_\mu \vert V \vert ^{1/2}$ is isospectral to $\mathcal {V}_\mu$, so its eigenvalues are given by Eq. (6), one can easily see, using Lemma 11 again, that

20 $$\begin{aligned} \limsup \limits _{\mu \rightarrow \infty } \mu ^{\beta ^*-\delta } \left\| V^{1/2} \mathfrak {F}_\mu ^\dagger \mathfrak {F}_\mu \vert V \vert ^{1/2}\right\| _{\mathrm {op}} = 0\,, \end{aligned}$$

for any $\delta >0$. Finally, by definition of $s^*$ (see Eq. (11)), we get for any $\delta >0$ that

21 $$\begin{aligned} \limsup \limits _{\mu \rightarrow \infty } \mu ^{\min (s^*,2)/2-\delta }\int \limits _{\mathbb {R}^3} \vert V(x)\vert \left( \frac{\sin (\sqrt{\mu } \vert x \vert )}{\sqrt{\mu }\vert x \vert }\right) ^2 \mathrm {d}x = 0\,. \end{aligned}$$

As the last ingredient we need the following Lemma, which provides a bound controlling $\Delta (p)$ in terms of $\Delta (\sqrt{\mu })$. Its proof is given in Sect. 2.2.

#### Lemma 13

Suppose $s^* > 1$ and let $u(p) = (4\pi )^{-1/2}$ be the constant function on the sphere $\mathbb {S} ^2$ and let

$$\begin{aligned} \hat{\varphi }(p) = \sqrt{4\pi }\mathfrak {F}V\mathfrak {F}_\mu ^\dagger u(p) = \frac{1}{(2\pi )^{3/2}} \int \limits _{ \mathbb {S} ^2} \hat{V}(p - \sqrt{\mu } q) \,\text {d}\omega (q)\,, \end{aligned}$$

where $\mathfrak {F}$ denotes the usual Fourier transform. Then

$$\begin{aligned} \Delta (p) = f(\mu )\left[ \hat{\varphi }(p) + \eta _\mu (p)\right] , \end{aligned}$$

for some function $f(\mu )$. The function $\eta _\mu$ satisfies

$$\begin{aligned} \limsup \limits _{\mu \rightarrow \infty } \mu ^{\beta ^* + \min (s^*,2)/4 - 1/2-\delta }\left\| \eta _\mu \right\| _{L^\infty } = 0  \text {and}  \limsup \limits _{\mu \rightarrow \infty } \mu ^{\beta ^* + \min (s^*,2)/2 - 1/2-\delta } \left| \eta _\mu (\sqrt{\mu }) \right| = 0 \end{aligned}$$

for any $$\delta > 0$$.

Note that $\hat{\varphi }(\sqrt{\mu }) = \sqrt{4\pi }\mathfrak {F}_\mu V\mathfrak {F}_\mu ^\dagger u(1) = e_\mu$. Now, combining this with Lemmas 11 and 12, we see that $\Delta (\sqrt{\mu }) = f(\mu ) e_\mu (1 + o(1))$, from which we conclude that

$$\begin{aligned} \Delta (p) = \frac{\hat{\varphi }(p) + \eta _\mu (p)}{e_\mu + \eta _\mu (\sqrt{\mu })} \Delta (\sqrt{\mu }) = \left[ 1 + \frac{\hat{\varphi }(p) - \hat{\varphi }(\sqrt{\mu })}{e_\mu } + \frac{\eta _\mu (p)}{e_\mu } \right] (1 + o(1)) \Delta (\sqrt{\mu })\,. \end{aligned}$$

Now, it is an easy computation to see $|\hat{\varphi }(p) - \hat{\varphi }(q)| \le C \mu ^{-1/2} |p-q|$ for all $p,q \in \mathbb {R}^3$. Thus

22 $$\begin{aligned} |\Delta (p)| \le C\left( 1 + \mu ^{\min (s^*,2)/2-1/2 + \delta }|p-\sqrt{\mu }| + \mu ^{\min (s^*,2)/4 - \beta ^* +1/2 + \delta }\right) \Delta (\sqrt{\mu }) \end{aligned}$$

for any $\delta >0$, again by means of Lemmas 11 and 12, assuming that *V* is admissible. So, we get the desired control on $\Delta (p)$ in terms of $\Delta (\sqrt{\mu })$.

The bound on $\eta _{\mu }(\sqrt{\mu })$ is effectively a bound on $\big \langle u \big \vert \mathfrak {F}_\mu ^\dagger V M_{\Delta , \mu }^{(\kappa )}V \mathfrak {F}_\mu \big \vert u \big \rangle$. (This will be clear from the proof.) For sufficiently large $\mu$ we have

23 $$\begin{aligned} \left| \left\langle u |\vert \mathfrak {F}_\mu ^\dagger V M_{\Delta , \mu }^{(\kappa )}V \mathfrak {F}_\mu |\vert u \right\rangle \right| \le C_\delta \mu ^{-\beta ^* - \min (s^*, 2)/2 + 1/2 + \delta } \end{aligned}$$

for any $\delta > 0$. This will be of importance in the perturbation argument in Proposition 15.

We are now able to prove the second and third equality in Lemma 7.

#### Proposition 14

Let *V* be an admissible potential. Then we have

$$\begin{aligned} m_\mu ^{(\kappa )}(\Delta ) = \sqrt{\mu } \left( \log \frac{\mu }{\Delta (\sqrt{\mu })}-2+\kappa \, \frac{\pi }{2} + \log (8) + o(1) \right) \end{aligned}$$

in the limit $\mu \rightarrow \infty$.

#### Proof

Computing the angular integral, and substituting $s = \pm \frac{p^2 - \mu }{\mu }$ we get

$$\begin{aligned} \begin{aligned} m_\mu ^{(\kappa )}(\Delta )&= \frac{\sqrt{\mu }}{2}\Bigg [ \int \limits _0^1 \left( \frac{\sqrt{1-s} - 1}{\sqrt{s^2 + x_-(s)^2}} + \frac{\sqrt{1 + s} - 1}{\sqrt{s^2 + x_+(s)^2}} - \frac{\sqrt{1-s}}{1-s + \kappa ^2} - \frac{\sqrt{1+s}}{1+s+\kappa ^2}\right) \,\text {d}s\\&\quad + \int \limits _0^1 \left( \frac{1}{\sqrt{s^2 + x_+(s)^2}} + \frac{1}{\sqrt{s^2 + x_-(s)^2}}\right) \,\text {d}s\\&\quad + \int \limits _1^\infty \left( \frac{\sqrt{1+s}}{\sqrt{s^2 + x_+(s)^2}} - \frac{\sqrt{1+s}}{1+s+\kappa ^2}\right) \,\text {d}s \Bigg ], \end{aligned} \end{aligned}$$

where $x_{\pm }(s) = \frac{\Delta (\sqrt{\mu }\sqrt{1\pm s})}{\mu }$. Now, using dominated convergence and $\Vert \Delta \Vert _{L^\infty } = o(\mu )$, it is easy to see that the first and last integrals converge to

$$\begin{aligned} \int \limits _0^1 \left( \frac{\sqrt{1-s} - 1}{s} + \frac{\sqrt{1 + s} - 1}{s} - \frac{\sqrt{1-s}}{1-s + \kappa ^2} - \frac{\sqrt{1+s}}{1+s+\kappa ^2}\right) \,\text {d}s \end{aligned}$$

and

$$\begin{aligned} \int \limits _1^\infty \left( \frac{\sqrt{1+s}}{s} - \frac{\sqrt{1+s}}{1+s+\kappa ^2}\right) \,\text {d}s\,, \end{aligned}$$

respectively, in the limit $\mu \rightarrow \infty$. For the middle integral we claim that

24 $$\begin{aligned} \int \limits _0^1 \left( \frac{1}{\sqrt{s^2 + x_\pm (s)^2}} - \frac{1}{\sqrt{s^2 + x_\pm (0)^2}}\right) \,\text {d}s \rightarrow 0 \quad \text {as} \quad \mu \rightarrow \infty \,. \end{aligned}$$

As in [9, 17] this is where we need both the Lipschitz–like bound on $\Delta$ (Lemma 9) and the bound controlling $\Delta (p)$ in terms of $\Delta (\sqrt{\mu })$ (Eq. (22)). In terms of $x_\pm$, Lemma 9 reads

25 $$\begin{aligned} |x_\pm (s) - x_\pm (0)| \le C\mu ^{-\delta _r} s\,. \end{aligned}$$

In terms of $x_\pm$, Eq. (22) reads

26 $$\begin{aligned} x_\pm (s) \le C(1 + \mu ^{\min (s^*,2)/2 +\delta } s + \mu ^{\min (s^*,2)/4 - \beta ^*+ 1/2 +\delta }) x_\pm (0)\,. \end{aligned}$$

Now, the integrand in Eq. (24) is bounded by

$$\begin{aligned} \frac{|x_\pm (s)^2 - x_\pm (0)^2|}{\sqrt{s^2 + x_\pm (s)^2}\sqrt{s^2 + x_\pm (0)^2}\left( \sqrt{s^2 + x_\pm (s)^2} + \sqrt{s^2 + x_\pm (0)^2}\right) }\,. \end{aligned}$$

We introduce a cutoff $\rho \in (0,1)$ and compute the integrals $\int _\rho ^1$ and $\int _0^\rho$. For the first integral we have

$$\begin{aligned} \begin{aligned}&\int \limits _\rho ^1 \frac{|x_\pm (s)^2 - x_\pm (0)^2|}{\sqrt{s^2 + x_\pm (s)^2}\sqrt{s^2 + x_\pm (0)^2}\left( \sqrt{s^2 + x_\pm (s)^2} + \sqrt{s^2 + x_\pm (0)^2}\right) } \,\text {d}s\\&\quad \le \ C \mu ^{-\delta _r} \int _\rho ^1 \frac{1}{s} \frac{x_\pm (s) + x_\pm (0)}{\sqrt{s^2 + x_\pm (s)^2} + \sqrt{s^2 + x_\pm (0)^2}} \,\text {d}s\\&\quad \le \ C \mu ^{-\delta _r} |\log \rho |\,. \end{aligned} \end{aligned}$$

which vanishes for any $\rho \gg \exp \left( -\mu ^{\delta _r}\right)$, in particular for $\rho = \mu ^{-N}$ for suitable $N > 0$, which we choose here. For the second integral we have

$$\begin{aligned} \begin{aligned}&\int \limits _0^\rho \frac{|x_\pm (s)^2 - x_\pm (0)^2|}{\sqrt{s^2 + x_\pm (s)^2}\sqrt{s^2 + x_\pm (0)^2}\left( \sqrt{s^2 + x_\pm (s)^2} + \sqrt{s^2 + x_\pm (0)^2}\right) } \,\text {d}s\\&\quad \le \ C \int \limits _0^\rho \mu ^{-\delta _r} \left( 1 + \mu ^{\min (s^*,2)/4 - \beta ^*+1/2+ \delta } + \mu ^{\min (s^*,2)/2+\delta } s\right) \frac{x_\pm (0)}{\sqrt{x_\pm (0)^2 + s^2}\left( s + \sqrt{x_\pm (0)^2 + s^2}\right) } \,\text {d}s\\&\quad \le \ C \mu ^{\min (s^*,2)/4 - \beta ^* - \delta _r +1/2+ \delta } \int \limits _0^\rho \frac{x_\pm (0)}{\sqrt{x_\pm (0)^2 + s^2}\left( s + \sqrt{x_\pm (0)^2 + s^2}\right) } \,\text {d}s\\&\quad \le \ C\mu ^{\min (s^*,2)/4 - \beta ^* - \delta _r + 1/2 + \delta }\,. \end{aligned} \end{aligned}$$

Note that for $r=2$, we have $\delta _{r=2} = 3/20$ and thus $\beta ^* - \min (s^*,2)/4-1/2+ 3/20 >0$ for any $s^* > 7/5$ (see Remark 2). Also, optimizing this expression in the allowed *r*’s gives the assumption $r > f(s^*)$ given in Remark 2. Therefore, also this second integral vanishes as desired by choosing $0< \delta < \beta ^* - \min (s^*,2)/4- 7/20$. We conclude that

$$\begin{aligned} \begin{aligned} m_\mu ^{(\kappa )}(\Delta )&= \frac{\sqrt{\mu }}{2}\Bigg [ \int \limits _0^1 \left( \frac{\sqrt{1-s} - 1}{s} + \frac{\sqrt{1 + s} - 1}{s} - \frac{\sqrt{1-s}}{1-s + \kappa ^2} - \frac{\sqrt{1+s}}{1+s+\kappa ^2}\right) \,\text {d}s \\&\quad + \int \limits _0^1 \frac{2}{\sqrt{s^2 + \left( \frac{\Delta (\sqrt{\mu })}{\mu }\right) ^2}} \,\text {d}s + \int _1^\infty \left( \frac{\sqrt{1+s}}{s} - \frac{\sqrt{1+s}}{1+s+\kappa ^2}\right) \,\text {d}s + o(1) \Bigg ]. \end{aligned} \end{aligned}$$

This may be computed (perhaps most easily by adding and subtracting the corresponding integral with $\kappa = 0$) as

$$\begin{aligned} m_\mu ^{(\kappa )} = \sqrt{\mu } \left( \log \frac{\mu }{\Delta (\sqrt{\mu })} - 2 + \log (8) + \kappa \frac{\pi }{2} + o(1)\right) . \end{aligned}$$

$$\square $$

We conclude by showing the third equality of Lemma 7.

#### Proposition 15

Let *V* be an admissible potential. Then

$$\begin{aligned} \frac{m_\mu ^{(\kappa )}(\Delta )}{\sqrt{\mu }} = - \frac{\pi }{2\sqrt{\mu }b_\mu ^{(\kappa )}} + o(1)\,. \end{aligned}$$

#### Proof

Recall that, by the Birman–Schwinger principle the lowest eigenvalue of $B_{\Delta ,\mu }$ is $-1$. Using the decomposition in Eq. (14) and the bound in Eq. (19) we get that

$$\begin{aligned} -1 = \lim \limits _{\mu \rightarrow \infty } m_\mu ^{(\kappa )}(\Delta ) \, \mathrm {inf\, spec} \left( V^{1/2} {\mathfrak {F}_{\mu }}^\dagger {\mathfrak {F}_{\mu }} \vert V \vert ^{1/2} \right) = \lim \limits _{\mu \rightarrow \infty } m_\mu ^{(\kappa )}(\Delta ) e_\mu \,. \end{aligned}$$

Now, since $s^* >7/5$ we have that $\vert \sqrt{\mu }e_\mu \vert \le C\mu ^{-2/5}$ by Lemma 11 (recall Eqs. (6) and (21)). Thus, by Proposition 14 we conclude that $\Delta (\sqrt{\mu })$ is exponentially small (in some positive power of $\mu$) as $\mu \rightarrow \infty$.

To obtain the next order in the expansion of $m_\mu (\Delta )$, we note that $1+V^{1/2} M_{\Delta ,\mu }^{(\kappa )} \vert V \vert ^{1/2}$ is invertible for $\mu$ large enough by means of Equation (19). We can thus factorize the Birman–Schwinger operator (14) as

$$\begin{aligned} 1 + B_{\Delta ,\mu } = \left( 1 + V^{1/2}M_{\Delta , \mu }^{(\kappa )}|V|^{1/2}\right) \left( 1 + \frac{m_\mu ^{(\kappa )}(\Delta )}{1 + V^{1/2}M_{\Delta ,\mu }^{(\kappa )}|V|^{1/2}} V^{1/2} \mathfrak {F}_\mu ^\dagger \mathfrak {F}_\mu |V|^{1/2}\right) \,. \end{aligned}$$

Because $B_{\Delta , \mu }$ has $-1$ as its lowest eigenvalue by the Birman–Schwinger principle, we conclude that, for $\mu$ large enough, the self–adjoint operator

$$\begin{aligned} T_{\Delta , \mu } := m_\mu ^{(\kappa )}(\Delta ) \mathfrak {F}_\mu |V|^{1/2} \frac{1}{1 + V^{1/2}M_{\Delta ,\mu }^{(\kappa )}|V|^{1/2}} V^{1/2}\mathfrak {F}_\mu ^\dagger \end{aligned}$$

acting on $L^2( \mathbb {S} ^2)$ has $-1$ as its lowest eigenvalue since it is isospectral to the right–most operator above. (This follows from the fact that for operators *A*, *B* the operators *AB* and *BA* have the same spectrum apart from possibly at 0. See also the argument around Eq. (33) in [14] as well as around Eqs. (30) and (47) in [9].)

To leading order $T_{\Delta , \mu }$ is proportional to $\mathcal {V}_\mu$. Since the constant function $u(p) = (4\pi )^{-1/2}$ on $\mathbb {S} ^2$ is the unique eigenvector of $\mathcal {V}_\mu$ with lowest eigenvalue, this is true also for $T_{\Delta , \mu }$ whenever $\mu$ is large enough.

To find the lowest eigenvalue (which is $-1$) we expand the geometric series to first order and employ first order perturbation theory. This is completely analogous to the arguments in [9] and Eq. (34) in [14]. We obtain

27 $$\begin{aligned} \frac{1}{\sqrt{\mu }} m_\mu ^{(\kappa )}(\Delta ) = \frac{-1}{\mu ^{1/2} e_\mu - \mu ^{1/2} \big \langle u \big \vert \mathfrak {F}_\mu V M_{\Delta ,\mu }^{(\kappa )} V \mathfrak {F}_\mu ^\dagger \big \vert u \big \rangle + O(\mu ^{-3\beta ^* + 3/2+\delta })} \end{aligned}$$

for any $\delta >0$ (recall Eqs. (19), (20) and (23)). The error term in Eq. (27) is twofold. The first part comes from the expansion of the geometric series. The second part comes from first order perturbation theory using the bounds

$$\begin{aligned} \vert \sqrt{\mu } e_\mu \vert \ge c_\delta \mu ^{-\min (s^*,2)/2+1/2-\delta }\quad \text {and} \quad \big \vert \mu ^{1/2}\big \langle u \big \vert \mathfrak {F}_\mu V M_{\Delta ,\mu }^{(\kappa )} V \mathfrak {F}_\mu ^\dagger \big \vert u \big \rangle \big \vert \le C_\delta \mu ^{-\beta ^* - \min (s^*, 2)/2 +1 + \delta } \end{aligned}$$

for any $\delta >0$ from Lemma 12 and Eq. (23). The error from the series expansion is of order $O(\mu ^{-3\beta ^* + 3/2+\delta })$ and the error from the perturbation argument is of order $O(\mu ^{-2\beta ^*-\min (s^*,2)/2 + 3/2+\delta })$ and is hence dominated by the expansion of the geometric series, since $\beta ^* \le \min (s^*,2)/2$.

Now, we need to show that $\mathfrak {F}_\mu V M_{\Delta ,\mu }^{(\kappa )} V \mathfrak {F}_\mu ^\dagger$ is close to $\mathcal {W}_\mu ^{(\kappa )}$, when evaluated in $\left\langle u |\vert \cdots |\vert u \right\rangle$. Therefore, considering their difference, we split the involved radial *p*-integral according to $|p| \le \mu ^N$ and $|p| > \mu ^N$ for some large $N >0$. The second part is clearly bounded by, e.g., $C \mu ^{-N/2}$. For the first part, we have $\Delta (p) \le C \mu ^N \Delta (\sqrt{\mu })$ by Eq. (22). Using this in combination with the fact that $\Delta (\sqrt{\mu })$ is exponentially small, we find, by dominated convergence and Lipschitz continuity of the involved angular integrals (cf. Eq. (35) in [9] and Eq. (36) in [14]), that this part is bounded by $C_D \mu ^{-D}$ for any $D > 0$. Since $N>0$ was arbitrary, we conclude that

28 $$\begin{aligned} \big \vert \big \langle u \big \vert \mathfrak {F}_\mu V M_{\Delta ,\mu }^{(\kappa )} V \mathfrak {F}_\mu ^\dagger - \mathcal {W}_\mu ^{(\kappa )} \big \vert u\big \rangle \big \vert \le C_D \mu ^{-D} \end{aligned}$$

for any $D>0$. Thus, by combining Eq. (23) and (28) (recall Eqs. (9) and 10) we get

29 $$\begin{aligned} \big \vert \big \langle u \big \vert \mathcal {W}_\mu ^{(\kappa )} \big \vert u \big \rangle \big \vert \le C_\delta \mu ^{-\beta ^* - \min (s^*,2)/2 +1/2 + \delta } \end{aligned}$$

for any $\delta > 0$. (In particular $b_\mu ^{(\kappa )} < 0$ for large $\mu$. This was also shown in [14].)

In particular, combining Eqs. (27), (28) and (29), we get again by a perturbation theory argument that

$$\begin{aligned} \frac{1}{\sqrt{\mu }} m_\mu ^{(\kappa )}(\Delta ) = - \frac{\pi }{2 \sqrt{\mu } b_\mu ^{(\kappa )}} + O(\mu ^{-3\beta ^* + \min (s^*,2)+1/2+\delta })\,, \end{aligned}$$

for any $\delta > 0$. Since $3\beta ^* - \min (s^*,2) -1/2 > 0$ we conclude the desired. $\square$

### Proofs of Auxiliary Lemmas

In this Subsection, we prove the auxiliary Lemmas 8, 9, and 13.

#### Proof of Lemma 8

First we show

30 $$\begin{aligned} \left\| \alpha \right\| _{H^1}^2 \le C \left\| \alpha \right\| _{L^2}^2 + C \mu ^{3/2}. \end{aligned}$$

Since $V\in L^{3/2}({\mathbb {R}^3})$ we have by Sobolev’s inequality [18,  Thm. 8.3] $\inf {{\,\mathrm{spec}\,}}\left( \frac{p^2}{2} + V\right) > -\infty$. Thus, using $\sqrt{1-4x^2}\le 1 - 2x^2$ and $\hat{\alpha }\le 1/2$ we get

$$\begin{aligned} \mathcal {F}(\alpha )&= \frac{1}{2} \int \limits _{\mathbb {R}^3} |p^2 - \mu | \left( 1 - \sqrt{1 - 4\hat{\alpha }(p)^2}\right) \,\text {d}p + \int \limits _{\mathbb {R}^3}V(x) |\alpha (x)|^2 \,\text {d}x\\&\ge \int \limits _{\mathbb {R}^3} (p^2 - \mu ) \hat{\alpha }(p)^2 \,\text {d}p + \int \limits _{\mathbb {R}^3} V(x)|\alpha (x)|^2 \,\text {d}x\\&= \left\langle \alpha |\vert \frac{p^2}{2} + V |\vert \alpha \right\rangle + \int \limits _{\mathbb {R}^3} \left( \frac{p^2}{2} - \mu \right) \hat{\alpha }(p)^2 \,\text {d}p\\&\ge \frac{1}{4}\left\| \alpha \right\| _{H^1}^2 - C \left\| \alpha \right\| _{L^2}^2 + \int \limits _{\mathbb {R}^3} \left( \frac{p^2}{4} - \mu - \frac{1}{4}\right) \hat{\alpha }(p)^2 \,\text {d}p\\&\ge \frac{1}{4}\left\| \alpha \right\| _{H^1}^2 - C\left\| \alpha \right\| _{L^2}^2 - \frac{1}{4} \int \limits _{\mathbb {R}^3} \left[ \frac{p^2}{4} - \mu - \frac{1}{4}\right] _{-} \,\text {d}p\\&\ge \frac{1}{4}\left\| \alpha \right\| _{H^1}^2 - C\left\| \alpha \right\| _{L^2}^2 - C \mu ^{3/2}\,, \end{aligned}$$

which gives the desired. Now we show that

$$\begin{aligned} \left\| \hat{\alpha }\mathbbm {1}_{\{|p| < t\}} \right\| _{L^2} \le C\left\| \hat{\alpha }\mathbbm {1}_{\{|p| > t\}} \right\| _{L^2} + \mu ^{\frac{3}{2} \delta - 1} \, \Vert \alpha \Vert _{H^1}, \end{aligned}$$

for $$t = \mu ^{\delta }$$ and $$0< \delta < 1/2$$.

To see this, we split the integrals in the functional $\mathcal {F}$ according to small or large momentum *p* and compute

$$\begin{aligned} \mathcal {F}(\alpha )&= \frac{1}{2} \int \limits _{\mathbb {R}^3} |p^2 - \mu | \left( 1 - \sqrt{1 - 4\hat{\alpha }(p)^2}\right) \,\text {d}p + \int \limits _{\mathbb {R}^3} V(x) |\alpha (x)|^2 \,\text {d}x\\&\ge \int \limits _{|p|< t} |p^2 - \mu | \hat{\alpha }(p)^2 \,\text {d}p + \int \limits _{|p|> t} |p^2 - \mu | \hat{\alpha }(p)^2 \,\text {d}p \\&\quad + \frac{1}{(2\pi )^{3/2}} \iint \limits _{\mathbb {R}^3\times \mathbb {R}^3} \hat{\alpha }(p) \hat{V}(p - q) \hat{\alpha }(q) \,\text {d}p \,\text {d}q \\&\ge \mu \left\| \hat{\alpha }\mathbbm {1}_{\{|p|< t\}} \right\| _{L^2}^2 - \left\| p^2\hat{\alpha }\mathbbm {1}_{\{|p|< t\}} \right\| _{L^2}^2 + \left\langle \hat{\alpha }\mathbbm {1}_{\{|p|> t\}} |\vert p^2 + V |\vert \hat{\alpha }\mathbbm {1}_{\{|p|> t\}} \right\rangle - \mu \left\| \hat{\alpha }\mathbbm {1}_{\{|p|> t\}} \right\| _{L^2}^2 \\&\quad + \frac{1}{(2\pi )^{3/2}} \left[ \iint \limits _{|p|, |q|< t}  \hat{\alpha }(p) \hat{V}(p - q) \hat{\alpha }(q) \,\text {d}p \,\text {d}q + 2 \iint \limits _{|p| < t, |q|>t} \hat{\alpha }(p) \hat{V}(p - q) \hat{\alpha }(q) \,\text {d}p \,\text {d}q \right] . \end{aligned}$$

Note that, again by Sobolev’s inequality [18,  Thm. 8.3], we have

$$\begin{aligned} \left\langle \hat{\alpha }\mathbbm {1}_{\{|p|> t\}} |\vert p^2 + V |\vert \hat{\alpha }\mathbbm {1}_{\{|p|> t\}} \right\rangle \ge - C\left\| \hat{\alpha }\mathbbm {1}_{\{|p| > t\}} \right\| _{L^2}^2\,. \end{aligned}$$

Moreover, by application of Young’s inequality [18,  Thm. 4.2] we obtain

$$\begin{aligned} \begin{aligned} \int \limits _{|p|< t} \int \limits _{|q|< t} \hat{\alpha }(p) \hat{V}(p - q) \hat{\alpha }(q) \,\text {d}p \,\text {d}q&\ge - \Vert \hat{V} \Vert _{L^3}\left\| \hat{\alpha }\mathbbm {1}_{\{|p|< t\}} \right\| _{L^{6/5}}^2 \\&\ge - C \left( t^3\right) ^{2\cdot (5/6 - 1/2)} \left\| \hat{\alpha }\mathbbm {1}_{\{|p|< t\}} \right\| _{L^2}^2 \\&= - C \mu ^{2\delta } \left\| \hat{\alpha }\mathbbm {1}_{\{|p| < t\}} \right\| _{L^2}^2 \end{aligned} \end{aligned}$$

and

$$\begin{aligned} \begin{aligned} \int \limits _{|p|< t} \int \limits _{|q|> t} \hat{\alpha }(p) \hat{V}(p - q) \hat{\alpha }(q) \,\text {d}p \,\text {d}q&\ge - \left\| \hat{\alpha }\mathbbm {1}_{\{|p|< t\}} \right\| _{L^1} \Vert \hat{V} \Vert _{L^3} \left\| \hat{\alpha }\mathbbm {1}_{\{|p| > t\}} \right\| _{L^{3/2}} \\&\ge - C t^{3/2} \left\| \alpha \right\| _{H^1} \left\| \hat{\alpha }\mathbbm {1}_{\{|p|< t\}} \right\| _{L^2} \\&= - C \mu ^{3\delta /2} \left\| \alpha \right\| _{H^1} \left\| \hat{\alpha }\mathbbm {1}_{\{|p| < t\}} \right\| _{L^2}\,, \end{aligned} \end{aligned}$$

where we used that $\left\| \hat{g} \right\| _{L^{3/2}} \le C \left\| g \right\| _{H^1}$. Thus we arrive at

$$\begin{aligned} \mathcal {F}(\alpha ) \ge c \mu \left\| \hat{\alpha }\mathbbm {1}_{\{|p|< t\}} \right\| _{L^2}^2 - C_1 \mu ^{3\delta /2} \left\| \alpha \right\| _{H^1} \left\| \hat{\alpha }\mathbbm {1}_{\{|p| < t\}} \right\| _{L^2} - C_2 \mu \left\| \hat{\alpha }\mathbbm {1}_{\{|p| > t\}} \right\| _{L^2}^2, \end{aligned}$$

where we absorbed all non–leading terms in these. This is a second degree polynomial in $\left\| \hat{\alpha }\mathbbm {1}_{\{|p| < t\}} \right\| _{L^2}$ and thus the value of $\left\| \hat{\alpha }\mathbbm {1}_{\{|p| < t\}} \right\| _{L^2}$ lies between the roots, i.e.

$$\begin{aligned} \begin{aligned} \left\| \hat{\alpha }\mathbbm {1}_{\{|p| < t\}} \right\| _{L^2}&\le \frac{ C_1 \mu ^{3\delta /2} \left\| \alpha \right\| _{H^1} + \sqrt{C_1^2\mu ^{3\delta } \left\| \alpha \right\| _{H^1}^2 + 4 c \, C_2 \mu ^2 \left\| \hat{\alpha }\mathbbm {1}_{\{|p|> t\}} \right\| _{L^2}^2} }{2c\mu } \\&\le C \left\| \hat{\alpha }\mathbbm {1}_{\{|p| > t\}} \right\| _{L^2} + C \mu ^{3\delta /2 - 1} \left\| \alpha \right\| _{H^1}. \end{aligned} \end{aligned}$$

From the estimate

$$\begin{aligned} \left\| \hat{\alpha }\mathbbm {1}_{\{|p|> t\}} \right\| _{L^2}^2 = \int \limits _{|p|> t} \hat{\alpha }(p)^2 \,\text {d}p \le \int \limits _{|p| > t} \hat{\alpha }(p)^2 \frac{1 + p^2}{1 + t^2}\,\text {d}p \le \frac{1}{1 + t^2}\left\| \alpha \right\| _{H^1}^2 \le C \mu ^{-2\delta }\left\| \alpha \right\| _{H^1}^2\,, \end{aligned}$$

we conclude that

$$\begin{aligned} \left\| \alpha \right\| _{L^2}^2 = \left\| \hat{\alpha }\mathbbm {1}_{\{|p| < t\}} \right\| _{L^2}^2 + \left\| \hat{\alpha }\mathbbm {1}_{\{|p| > t\}} \right\| _{L^2}^2 \le C \left( \mu ^{-2\delta } + \mu ^{3\delta - 2}\right) \left\| \alpha \right\| _{H^1}^2. \end{aligned}$$

Choosing the optimal $\delta = 2/5$ we get $\left\| \alpha \right\| _{L^2} \le C \mu ^{-2/5} \left\| \alpha \right\| _{H^1}$, which, in combination with Eq. (30), yields

$$\begin{aligned} \left\| \alpha \right\| _{H^1}^2 \le C \mu ^{-4/5} \left\| \alpha \right\| _{H^1}^2 + \mu ^{3/2}. \end{aligned}$$

Hence $\left\| \alpha \right\| _{H^1} \le C\mu ^{3/4}$ and thus also $\left\| \alpha \right\| _{L^2} \le C \mu ^{-2/5} \left\| \alpha \right\| _{H^1} \le C \mu ^{7/20}$ for sufficiently large $\mu$. $\square$

We now turn to the proof of Lemma 9.

#### Proof of Lemma 9

Let $t = \frac{5}{2} - \frac{3}{r}$. Then we have

$$\begin{aligned} \left\| \Delta \right\| _{L^\infty } \le C \left\| V\alpha \right\| _{L^1} \le C \left\| V \right\| _{L^{r}} \left\| \alpha \right\| _{L^{{r}'}} \le C \left\| \alpha \right\| _{L^2}^t \left\| \alpha \right\| _{L^6}^{1-t} \le C \mu ^{\frac{15-8t}{20}} = C \mu ^{\frac{24-5r}{20r}} \end{aligned}$$

by Sobolev’s inequality [18,  Thm. 8.3]. For the difference note that $\Delta (p) - \Delta (q)$ is (proportional to) the Fourier transform of $V(x)\left( 1 - {\mathrm {e}}^{\mathrm {i}(p-q)\cdot x}\right) \alpha (x)$. Then

$$\begin{aligned} \left\| V(x)\left( 1 - {\mathrm {e}}^{\mathrm {i}(p-q)x}\right) \right\| _{L^{r}}^{r} = \int \limits _{\mathbb {R}^3} |V(x)|^{r} \left| 1 - {\mathrm {e}}^{\mathrm {i}(p-q)\cdot x} \right| ^{r} \,\text {d}x \le C \int \limits _{\mathbb {R}^3} |V(x)|^{r} |p-q|^{r} |x|^{r} \,\text {d}x. \end{aligned}$$

Using radiality of $\Delta$, the same argument as before gives the desired. $\square$

Finally, we give the proof of Lemma 13.

#### Proof of Lemma 13

Recall from the factorization of the Birman–Schwinger operator in the proof of Proposition 15, that the self–adjoint operator

$$\begin{aligned} m_\mu ^{(\kappa )}(\Delta ) \mathfrak {F}_\mu |V|^{1/2} \frac{1}{1 + V^{1/2}M_{\Delta ,\mu }^{(\kappa )}|V|^{1/2}} V^{1/2}\mathfrak {F}_\mu ^\dagger \end{aligned}$$

acting on $L^2( \mathbb {S} ^2)$ has $-1$ as its lowest eigenvalue and $u(p) = (4\pi )^{-1/2}$ is the unique eigenvector with lowest eigenvalue for $\mu$ large enough. Hence, one can easily see that

$$\begin{aligned} \frac{1}{1 + V^{1/2}M_{\Delta ,\mu }^{(\kappa )}|V|^{1/2}} V^{1/2} \mathfrak {F}_\mu ^\dagger u \end{aligned}$$

is an eigenvector of $B_{\Delta ,\mu }$ for the lowest eigenvalue and thus proportional to $V^{1/2}\alpha$. By expanding $\frac{1}{1+x} = 1 - \frac{x}{1+x}$ we conclude that $\Delta = f(\mu )[\hat{\varphi }+ \eta _\mu ]$, where

$$\begin{aligned} \eta _\mu = - \sqrt{4\pi }\mathfrak {F}|V|^{1/2} \frac{V^{1/2} M_{\Delta ,\mu }^{(\kappa )} |V|^{1/2}}{1 + V^{1/2} M_{\Delta ,\mu }^{(\kappa )} |V|^{1/2}} V^{1/2} \mathfrak {F}_\mu ^\dagger u\,, \end{aligned}$$

which can be bounded easily as

$$\begin{aligned} \left\| \eta _\mu \right\| _{L^\infty } \le C \left\| V \right\| _{L^1}^{1/2} \left\| V^{1/2} M_{\Delta ,\mu }^{(\kappa )} |V|^{1/2} \right\| _{\mathrm {op}} \left\| V^{1/2} \mathfrak {F}_\mu ^\dagger u \right\| _{L^2}\,. \end{aligned}$$

For $\vert p \vert = \sqrt{\mu }$, we first note that $\hat{\varphi }(\sqrt{\mu }) = \sqrt{4\pi }\mathfrak {F}_\mu V \mathfrak {F}_\mu ^\dagger u(1) = e_\mu$. Similarly, since $\eta _\mu$ is radial, we have that

$$\begin{aligned} \begin{aligned} \eta _\mu (\sqrt{\mu })&= \frac{1}{4\pi } \int \limits _{ \mathbb {S} ^2} \eta _\mu (\sqrt{\mu }q) \,\text {d}\omega (q) = - \left\langle u |\vert \mathfrak {F}_\mu |V|^{1/2} \frac{V^{1/2} M_{\Delta ,\mu }^{(\kappa )} |V|^{1/2}}{1 + V^{1/2} M_{\Delta ,\mu }^{(\kappa )} |V|^{1/2}} V^{1/2} \mathfrak {F}_\mu ^\dagger |\vert u \right\rangle \end{aligned} \end{aligned}$$

and we can thus bound

$$\begin{aligned} \left| \eta _\mu (\sqrt{\mu }) \right| \le C \left\| V^{1/2} M_{\Delta ,\mu }^{(\kappa )} |V|^{1/2} \right\| _{\mathrm {op}} \left\| |V|^{1/2} \mathfrak {F}_\mu ^\dagger u \right\| _{L^2}^2. \end{aligned}$$

It remains to check that

$$\begin{aligned} \left\| |V|^{1/2} \mathfrak {F}_\mu ^\dagger u \right\| _{L^2}^2 = C \int \limits _{\mathbb {R}^3} |V(x)| \left| \int \limits _{ \mathbb {S} ^2} e^{i\sqrt{\mu }p\cdot x} \frac{1}{\sqrt{4\pi }}\,\text {d}\omega (p) \right| ^2 \mathrm {d}x= C \int \limits _{\mathbb {R}^3} |V(x)|\left( \frac{\sin \sqrt{\mu }|x|}{\sqrt{\mu }|x|}\right) ^2 \mathrm {d}x\,. \end{aligned}$$

Now the claim follows by application of Eqs. (19) and (21). $\square$

## Data Availability

Data sharing is not applicable to this article as no datasets were generated or analyzed during the current study.
